# Laryngopharyngeal reflux and psychological distress: a vicious cycle worth investigating

**DOI:** 10.1192/j.eurpsy.2026.10647

**Published:** 2026-07-31

**Authors:** C. Recano, M. R. Barillari, G. M. Giordano, G. Costa, E. Caporusso, F. Giumello, S. Tolone, J. Lechien, A. Maniaci, C. M. Chiesa-Estomba, M. Mayo Yanez, A. Nacci, A. Mucci, S. Galderisi, L. Bastiani

**Affiliations:** 1 Department of Psychiatry, “L. Vanvitelli” University; 2 Department of Mental and Physical Health and Preventive Medicine, “L. Vanvitelli” University, Napoli, Italy; 3 Laryngology Study Group of the Young-Otolaryngologists of the International Federations of Oto-Rhino-Laryngological Societies (YO-IFOS), Marseille, France; 4 Division of Phoniatrics and Audiology, “L. Vanvitelli”; 5 Department of Advanced Medical and Surgical Sciences, University of Campania Luigi Vanvitelli, napoli, Italy; 6 2 Laryngology Study Group of the Young-Otolaryngologists of the International Federations of Oto-Rhino-Laryngological Societies (YO-IFOS), Marseille, France; 7 Division of Laryngology and Bronchoesophagology, EpiCURA Hospital, University of Mons, Mons, Belgium; 8 Laryngology Study Group of the Young-Otolaryngologists of the International Federations of Oto-Rhino-Laryngological Societies (YO-IFOS), Marseille; 9 Department of Medicine and Surgery, University of Enna “Kore”, Enna; 10 Laryngology Study Group of the Young-Otolaryngologists of the International Federations of Oto-Rhino-Laryngological Societies (YO-IFOS), Marseille, Marseille, Italy; 11 Department of Otorhinolaryngology-Head and Neck Surgery, Hospital Universitario Donostia, San Sebastian; 12 Department of Otorhinolaryngology-Head and Neck Surgery, Hospital San Rafael (HSR), Coruna, Spain; 13 ENT, Audiology and Phoniatrics Unit, University of Pisa; 14 Epidemiology Section, CNR Institute of Clinical Physiology, Pisa, Italy

## Abstract

**Introduction:**

Laryngopharyngeal reflux (LPR) is a common disorder characterized by upper aerodigestive symptoms, with growing evidence suggesting that psychological distress may act as a trigger or exacerbating factor. However, findings remain inconsistent and mostly focused on GERD rather than LPR.

**Objectives:**

To investigate the correlation between laryngopharyngeal reflux (LPR) and psychological distress in adult Italian patients

**Methods:**

LPR was assessed using the Reflux Symptom Index (RSI), Reflux Finding Score (RFS), and 24-hour impedance-pH monitoring. Psychological distress was evaluated with the following clinical tools: the Hospital Anxiety and Depression Scale (HADS), the Hamilton Anxiety Rating Scale (HAM-A), the Hamilton Depression Rating Scale (HAM-D), the Impact of Event Scale-Revised (IES-R), the Insomnia Severity Index (ISI), and the Perceived Stress Scale-10 (PSS-10). Associations between RSI, RFS, and psychological scores were analyzed

**Results:**

A total of 45 LPR patients (Study Group, SG) and 29 healthy controls (CG) were included. Psychological assessments showed significantly higher distress in the SG compared to CG, with the only exception of the ISI. Anxiety levels were greater in LPR patients (HAM-A: 9.53 ± 5.8 vs. 6.79 ± 6.5; p = 0.025), as were anxious-depressive symptoms (HADS, p = 0.029). Depressive symptoms remained on average below the clinical threshold in both groups, though SG scores were close to the cut-off (6.89 ± 4.1 vs. 4.86 ± 5.1; p = 0.010). Perceived stress was significantly higher in the SG, reaching mild-to-moderate levels (21.62 ± 8.1 vs. 13.90 ± 5.5; p < 0.001). Moreover, RSI scores correlated positively with HAM-D, HADS, and HAM-A values.

**Conclusions:**

Patients with LPR showed significantly higher levels of psychological distress, particularly anxiety and perceived stress, compared with healthy controls. These findings suggest that psychological factors may play an important role in the management of LPR and should be taken into account in diagnostic and therapeutic pathways.

**Disclosure of Interest:**

None Declared

